# Use of intravenous valproate in acute manic: about a clinical case

**DOI:** 10.1192/j.eurpsy.2022.1042

**Published:** 2022-09-01

**Authors:** M. Suárez Gómez, A. Suárez Gómez, P. Rodrigues, S.S. Sánchez Rus, A. Matos-Pires

**Affiliations:** 1 ULSBA, Psychiatry, Beja, Portugal; 2 UCC, Community Health, Borba, Portugal; 3 Hospital Neuropsiquiátrico de Jaén, Psychiatry, Jaén, Spain

**Keywords:** Acute manic, Bipolar disorser type I, emergency, intravenous valproate

## Abstract

**Introduction:**

Acute mania can have behavioral effects such as agitation, being a frequent cause of presentation in the emergency department. Pharmacological treatments include mood stabilizers and atypical antipsychotics. Valproate is an effective drug. However, the intravenous formulation is relegated to other pathologies, such as epilepsy.

**Objectives:**

The objective was to review the use of intravenous valproate in acute mania in the literature and present its use through a clinical case.

**Methods:**

A clinical case using intravenous valproate to treat an episode of acute mania is described and the scientific literature of the last 5 years is reviewed.

**Results:**

A 43-year-old patient attended the emergency department with a diagnosis of bipolar disorder type I in manic episode with agitation, rejection of oral medication, brought in by the police due to risk of aggression against family members, who reported that the patient had stopped taking her usual medication with valproate 500 mg / 24h and quetiapine 200 mg / 24h threemonths ago. Due to the possibility of having intravenous valproate, it was decided to administer 300 mg intravenously, as well as haloperidol 5 mg intravenously, and hospitalization was decided. The patient had a favorable evolution, with no side effects to the medication, and oral treatment was started after 8 hours, with a good response. In the literature there are few studies in this regard, although the most of them approved the use of valproate as a loading dose in acute mania.

**Conclusions:**

Intravenous valproate is an effective, safe, and tolerated treatment in acute mania. More studies are needed to collect precise information.

**Disclosure:**

No significant relationships.

